# Salisbury Infirmary and Contributory Organisations

**Published:** 1924-03

**Authors:** L. S. Luckham

**Affiliations:** President Salisbury and District Infirmary and Hospital League. League Office, General Infirmary, Salisbury


### CORRESPONDENCE.

[Correspondence on all subjects is invited, but we cannot be responsible for the opinions expressed by our correspondents, who must give name and address as a guarantee of good faith, but not necessarily for publication. Correspondents are reminded that conciseness greatly facilitates early insertion.]

### Salisbury Infirmary and Contributory Organisations.

*To the Editor of* THE HOSPITAL AND HEALTH REVIEW.

Sir,—I understand from a friend who was at the Chichester meeting of the Regional Committee that it was there stated that our League was not co-operating with other contributory organisations, and that we paid 10s. 6d. only to other hospitals. I must confess I was considerably surprised to learn that such a misapprehension should exist, for we rather pride ourselves that we were probably the first to put forward a definite scheme of co-operation at a meeting held here on May 27, 1922. Unfortunately, we have not been able to get our neighbours to see that this suggested scheme is one that honestly makes an attempt to deal fairly with all the districts and hospitals in our area, and that it will get rid of the unfair conditions of support (or, rather, want of support) that at present exist.

A member of our League was taken into Winchester Hospital in 1922, and we established the precedent of asking for a note of his total cost, and paying for it, although there was some demur on the part of the Winchester authorities, who wished us to adopt the reciprocal plan of a free interchange of patients. Obviously, this would not be anything like so fair a plan as ours, for one hospital might be saddled with the cost of ten members against the cost of one. Our scheme entirely and fairly gets over this difficulty, and, in our opinion, is more equitable than the Winchester and Andover arrangement. It is true that we pay no more than the 10s. 6d. to Cottage Hospitals who do not co-operate and have no contributory scheme, as we find (as do other General Hospitals) that we finance the hospital needs of their districts to the extent of some hundreds of pounds yearly.

As regards the London Hospitals, our Committee has so far only paid the 10s. 6d. weekly, as at present our Infirmary finance is in such a parlous state that it is felt we cannot do more until this is remedied. The cost of London hospital patients is also much larger (in many cases double) than that of provincial hospitals, and I would suggest that the fair way of dealing with this—when it can be done—will be for provincial hospitals and leagues to pay the provincial cost only for members in London hospitals. Districts such as ours, largely agricultural, have not a wealthy public or highly paid artisans from whom to draw subscriptions and contributions. The spirit met with in our villages is splendid, and help is given ungrudgingly, but we must not ask them for more than their fair share of support. As these Leagues progress it may be possible for all to afford other benefits—as some already do—in the way of provision of nursing, etc. After all, the great thing for us to remember is that we are dealing with a limited charitable income, and that it is our duty, by friendly co-operation and interchange of ideas, to try to arrive at some result that will afford our artisan population the best possible hospital service such income can give.

It may not be out of place here to repeat the resolutions which were tentatively put forward from the Chair as a basis for discussion and consideration at our meeting on May 27, 1922. They ran as follows :—" That this Meeting is of the opinion (1) that a contributory scheme should be adopted throughout the area served by their hospitals where such does not already exist; (2) that the general hospitals concerned should be asked to agree upon boundaries; (3) that in each such hospital district the Cottage Hospitals be asked to form a branch under the name of that hospital—for instance, the Salisbury and District Infirmary and Hospital League Branch; (4) that all contributory members should have their maintenance contribution paid for them when an inmate of any hospital or convalescent home; (5) that the balance of funds available afterwards should at the end of every three or six months be allocated as follows :—To each hospital the total average cost of every such member using that hospital—less the maintenance already paid; any further balance to be divided on the same scale according to the number of patients."

L. S. LUCKHAM, M.R.C.S.,
President Salisbury and District Infirmary and Hospital League.

League Office,
General Infirmary, Salisbury.

[We learn that since this letter was written the Bridgwater Hospital Special Committee appointed to consider the subject has adopted the Salisbury Contributory Scheme in its entirety, and that an organisation is being constituted to put it into immediate operation.—ED. H. & H. R.]

